# GeneTrail 3: advanced high-throughput enrichment analysis

**DOI:** 10.1093/nar/gkaa306

**Published:** 2020-05-07

**Authors:** Nico Gerstner, Tim Kehl, Kerstin Lenhof, Anne Müller, Carolin Mayer, Lea Eckhart, Nadja Liddy Grammes, Caroline Diener, Martin Hart, Oliver Hahn, Jörn Walter, Tony Wyss-Coray, Eckart Meese, Andreas Keller, Hans-Peter Lenhof

**Affiliations:** Center for Bioinformatics, Saarland Informatics Campus, Saarland University, 66123 Saarbrücken, Germany; Center for Bioinformatics, Saarland Informatics Campus, Saarland University, 66123 Saarbrücken, Germany; Center for Bioinformatics, Saarland Informatics Campus, Saarland University, 66123 Saarbrücken, Germany; Center for Bioinformatics, Saarland Informatics Campus, Saarland University, 66123 Saarbrücken, Germany; Center for Bioinformatics, Saarland Informatics Campus, Saarland University, 66123 Saarbrücken, Germany; Center for Bioinformatics, Saarland Informatics Campus, Saarland University, 66123 Saarbrücken, Germany; Center for Bioinformatics, Saarland Informatics Campus, Saarland University, 66123 Saarbrücken, Germany; Chair for Clinical Bioinformatics, Saarland University, 66123 Saarbrücken, Germany; Department of Human Genetics, Saarland University, 66421 Homburg, Germany; Department of Human Genetics, Saarland University, 66421 Homburg, Germany; School of Medicine Office, Stanford University, Stanford, CA, USA; Department of Neurology and Neurological Sciences, Stanford University, Stanford, CA, USA; Department of Genetics, Saarland University, Saarbrücken D-66041, Germany; School of Medicine Office, Stanford University, Stanford, CA, USA; Department of Neurology and Neurological Sciences, Stanford University, Stanford, CA, USA; Department of Human Genetics, Saarland University, 66421 Homburg, Germany; Center for Bioinformatics, Saarland Informatics Campus, Saarland University, 66123 Saarbrücken, Germany; Chair for Clinical Bioinformatics, Saarland University, 66123 Saarbrücken, Germany; School of Medicine Office, Stanford University, Stanford, CA, USA; Department of Neurology and Neurological Sciences, Stanford University, Stanford, CA, USA; Center for Bioinformatics, Saarland Informatics Campus, Saarland University, 66123 Saarbrücken, Germany

## Abstract

We present GeneTrail 3, a major extension of our web service GeneTrail that offers rich functionality for the identification, analysis, and visualization of deregulated biological processes. Our web service provides a comprehensive collection of biological processes and signaling pathways for 12 model organisms that can be analyzed with a powerful framework for enrichment and network analysis of transcriptomic, miRNomic, proteomic, and genomic data sets. Moreover, GeneTrail offers novel workflows for the analysis of epigenetic marks, time series experiments, and single cell data. We demonstrate the capabilities of our web service in two case-studies, which highlight that GeneTrail is well equipped for uncovering complex molecular mechanisms. GeneTrail is freely accessible at: http://genetrail.bioinf.uni-sb.de.

## INTRODUCTION

Modern high-throughput technologies have revolutionized biomedical research by enabling comprehensive molecular profiling of biological systems. Methods like high-throughput sequencing, microarrays, or mass spectrometry are now routinely applied to generate huge multi-omics data sets.

Enrichment and network analysis procedures are an important class of computational methods designed for the analysis of these high-dimensional data sets with the major goal to gain novel insights into biological processes. In general, these approaches use statistical tests to determine if biological categories under investigation are deregulated. The most widely used methods in this context are Over-Representation Analysis (ORA) (1) and Gene Set Enrichment Analysis (GSEA) (2).

Over the years, a variety of tools for enrichment analysis have been published. Most of these tools focus on the analysis of specific omics data types. For example, DAVID (3), GSEA-P (4), and Webgestalt (5) have been developed for the analysis of gene expression data. DIANA-miRPath (6) or miEAA (7) provide workflows for enrichment analysis of miRNA data sets. GSEA-SNP (8) or i-GSEA4GWAS (9) can be used to test if SNPs that are associated with a disease phenotype are enriched in signaling pathways. LOLAweb (10) allows the user to study significant overlaps between genomic regions and predefined genomic annotations.

In addition to the approaches for individual omics types, several tools can analyze and integrate different omics data types, for example Enrichr (12), iPEAP (11), PaintOmics 3 (13), RAMONA (14), and our web service GeneTrail (15,16). A more detailed description and comparison of the different tools can be found in Supplement S1.

Since the rapid advancements of wet lab technologies enable the generation of more and more voluminous and complex data sets, computational analysis tools also have accordingly to be refined in order to deal with this progress. Amongst others, single-cell data sets with thousands of noisy and sparse samples pose new challenges concerning the developments of computational methods. To cope with the development of high-throughput technologies, we substantially extended the functionality of GeneTrail.

The third version of our web service provides rich functionality for the integrated analysis and interactive visualization of transcriptomic, miRNomic, proteomic and genomic data sets. It offers a powerful framework for enrichment and network analysis and a comprehensive collection of predefined biological processes and signaling pathways for 12 model organisms. On top of this, GeneTrail 3 provides novel workflows for the analysis of epigenetic marks, time series experiments, and single cell data. For all workflows, interactive visualizations assist the user in analyzing the results and thus help to facilitate their interpretation.

To demonstrate the capabilities of our web server, we present two case studies. First, we analyze a time-resolved gene expression data set of CD4+ T cells from human blood. The isolated T cells were in vitro activated and expression profiles were created at 2 h intervals from 0 to 24 h. In particular, we explore signaling pathways that exhibit altered expression patterns after T cell activation. In a second case study, we analyze a single cell data set of mouse microglia cells. In total, the data set contains gene expression profiles of 8330 individual cells from mice that belong to three different age groups: 3 month (2219 cells), 18 month (1998 cells), and 24 month (4113 cells). The major goal here is to study the hallmarks of aging.

## GENERAL FUNCTIONALITY AND NEW WORKFLOWS

Since the initial release of GeneTrail in 2007, our web service has been continuously maintained, refined, and extended. It provides a powerful framework for the identification of deregulated biological processes. Users can choose from a large collection of statistical tests to build custom pipelines. Currently, GeneTrail offers 15 tests to detect differentially expressed genes, proteins, or miRNAs, 11 methods to conduct enrichment analysis with different strategies to calculate *P*-values, and 9 methods for multiple testing correction. Based on this comprehensive analysis functionality, our web service can be applied to analyze a huge collection of biological processes and signaling pathways for 12 model organisms. For *Homo sapiens* alone, GeneTrail 3 provides nearly 65 000 biological categories for the analysis of genes and proteins and 33 000 for miRNAs (cf. Supplement S2). These include popular databases like GO (17), KEGG (18), and Reactome (19).

A variety of routines help to reduce the required user interaction by analyzing all uploaded data sets. Amongst others, GeneTrail automatically detects identifier types of the uploaded biological entities. Different properties of the data are utilized to preselect a suitable combination of statistical methods with sensible default parameters for all analyses. In combination with our interactive web interface and thorough documentation, this allows even non-expert users to carry out complex analyses of multi-omics data sets. For all workflows, we created interactive visualizations that range from a general overview of the data to an in-depth representation of specific results and thus help the users to interpret the results.

GeneTrail also offers a RESTful API that provides programmatic access to the entire functionality. This enables users to integrate our web service into third party workflows. It is also seamlessly integrated with its sister projects that provide additional analysis functionality. For example, NetworkTrail can be used to identify deregulated subgraphs in biological networks (20), RegulatorTrail (21) can help to detect influential transcriptional regulators, and DrugTargetInspector (22) or ClinOmicsTrail (23) can assist in the treatment stratification process of cancer patients.

Different workflows for the integrative analysis of transcriptomic, miRNomic, proteomic, and genomic data sets have already been described in previous publications (15,16). Hence, we discuss only the novel workflows for the analysis of epigenetic marks, time series experiments, and single cell data.

### Epigenomics workflow

The epigenomics workflow can be used to analyze histone marks, DNA methylation patterns, and open-chromatin regions with the major goal to uncover epigenetic mechanisms that control the natural and pathogenic processes under investigation.

Users can upload epigenetic data sets for different sample groups in form of BED files. For each sample group, GeneTrail analyzes the corresponding epigenetic marks and assigns chromatin states (active, poised, repressed, or no signal) to each individual gene. To this end, we employ a knowledge-based approach that considers specific combinations of epigenetic marks in the promoter, enhancer, and gene body regions of each gene. A detailed description can be found in Supplement S3. For each pair of sample groups, our web service then determines for each gene if there is a change of its chromatin state, e.g. if there is a transition from a poised state in the first group to an active state in the other group. Genes with the same behaviour are then combined into state transition groups, e.g. (poised → active). For each of these groups, over-representation analyses are carried out to identify biological processes, molecular functions, and cellular components that are enriched with the genes of this transition group.

**Figure 1.**
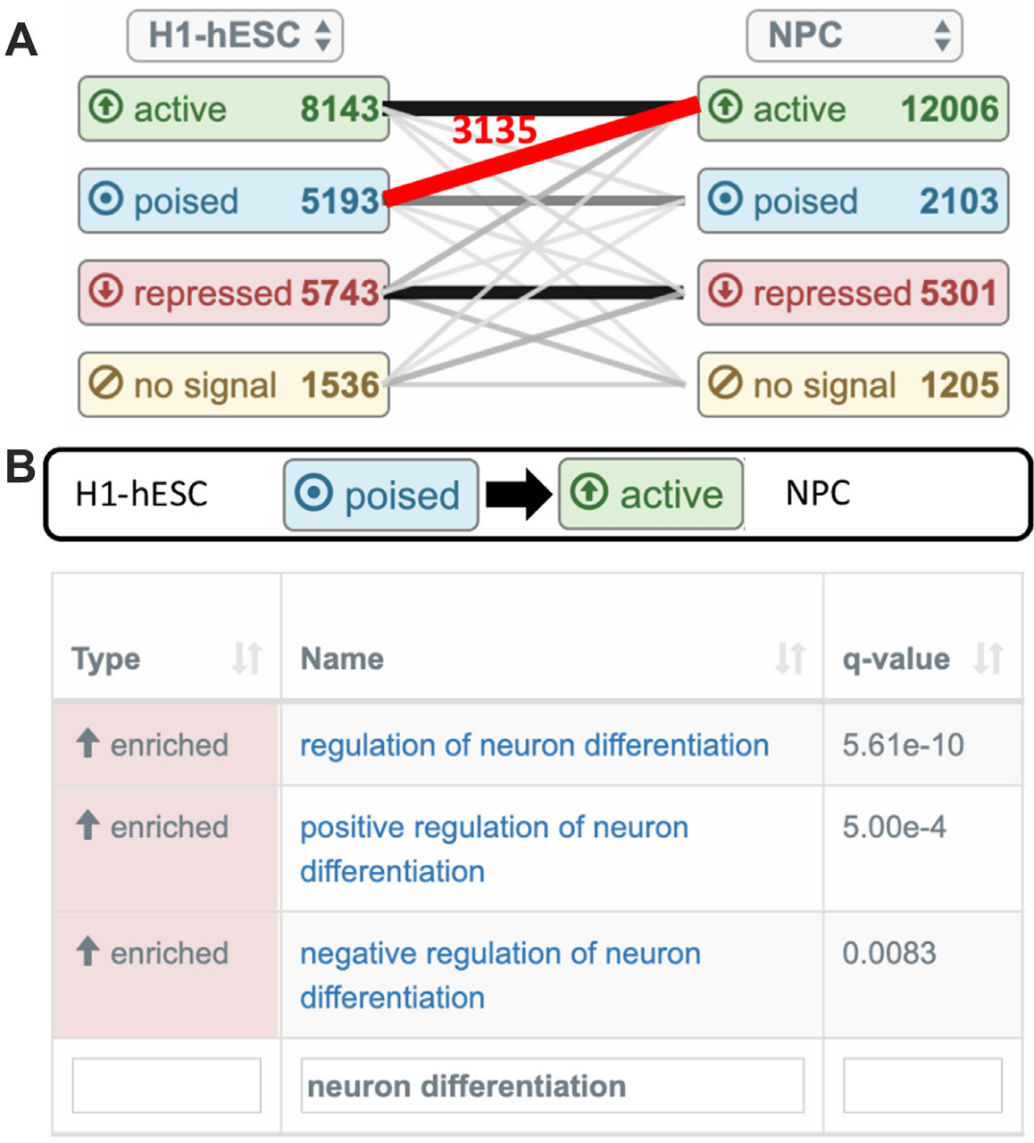
Example of an epigenomic analysis that is based on the following epigenetic marks of human embryonic stem cells (H1-hESC, Encode ENCSR938GXK) and neural progenitor cells (NPC, Encode ENCSR539JGB): H3K4me3, H3K9me3, H3K27ac, H3K27me3, H3K36me3. (**A**) Graph representation of chromatin state transitions. Here, vertices, which represent chromatin states, show the number of genes in a state and edges the respective transitions. The weight of each edge corresponds to the number of genes in this transition group. (**B**) Enrichment results of transition group (poised → active) (marked in red). The results have been filtered for categories that have the term ‘neuron differentiation’ in their name.

Finally, GeneTrail summarizes the results in a graph-like representation, whose vertices represent the different sample groups and states and whose edges represent the state transition groups (cf. Figure 1A). Users can select individual transitions via a mouse click on the corresponding edge, and GeneTrail then shows the associated enrichment results (cf. Figure 1B) that can be interactively explored.

### Time series workflow

The time series workflow provides functionality for the analysis of time-resolved expression data sets. Our web server first identifies clusters of biological entities, e.g. genes, with very similar expression time curves and then tests for each cluster which processes and signaling pathways are regulated by the members of the cluster.

Users can upload a time-resolved gene, protein, or miRNA expression data set in form of a white-space separated matrix. Our web service then carries out the following preprocessing and analysis steps (cf. Supplement S4). In the preprocessing step, all genes that show only limited expression changes over time are filtered out. Then a two-stage clustering approach is applied to identify groups of genes, miRNAs, or proteins with similar expression patterns. For this purpose, users can select a distance measure and a clustering algorithm from a variety of approaches. First, a strict clustering, which generates small groups with a high similarity between all members, is performed. The second clustering then combines similar clusters generated in the first step to so-called 'super-clusters'. In a last step, GeneTrail carries out over-representation analyses for all clusters and super-clusters in order to identify associated biological processes and signaling pathways.

**Figure 2.**
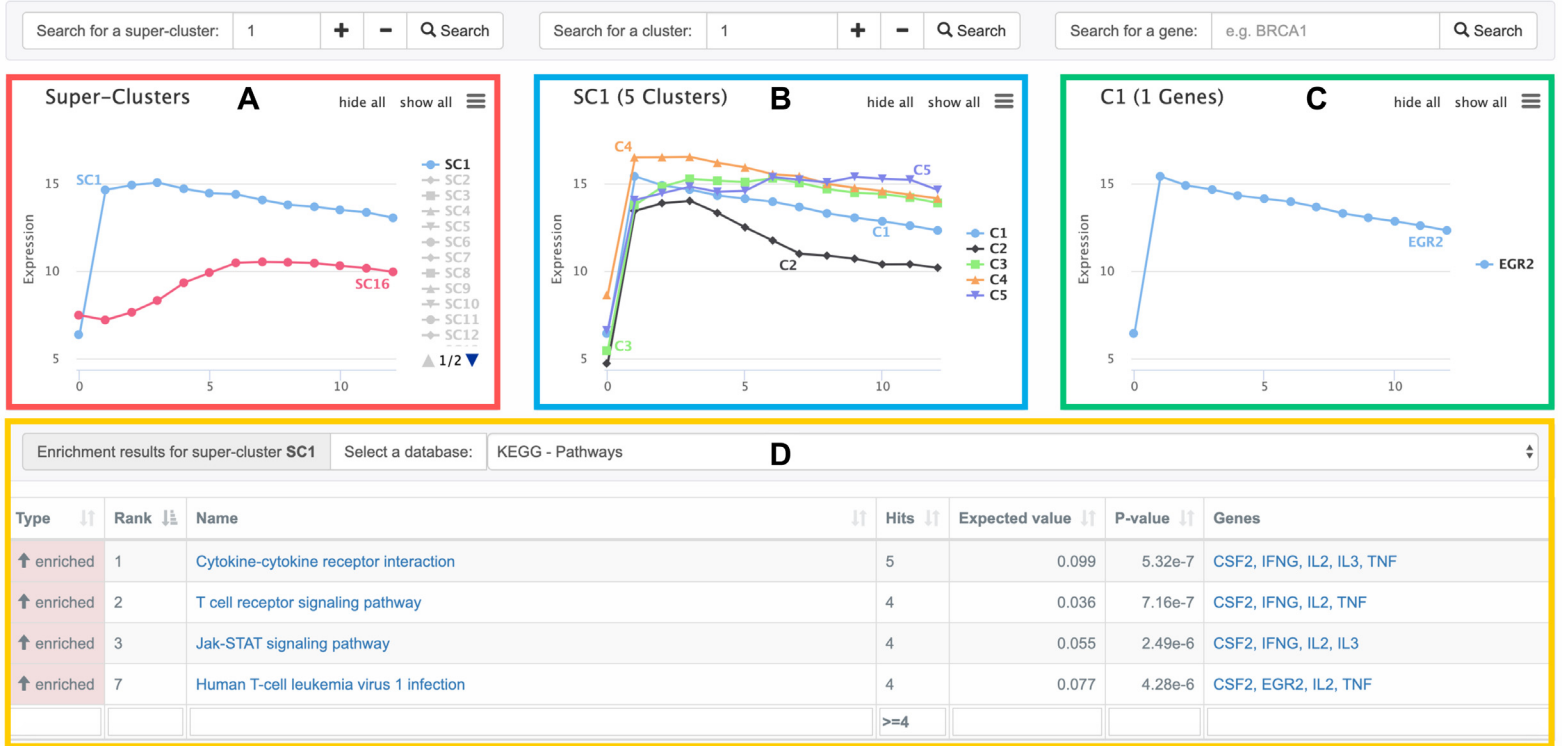
Screenshot of the time-series analysis results for CD4+ (GSE136625). (**A**) Overview of calculated super-clusters. Only mean expression curves of the super-clusters SC1 and SC16 are shown. (**B**) Mean time courses of all sub-clusters of SC1. (**C**) Member genes of cluster C1. (**D**) Filtered enrichment results (KEGG Pathways) of SC1.

The clustering and enrichment results are then presented in an interactive visualization that summarizes the different levels of information (cf. Figure 2). The small number of super-clusters shown on the left side represents a coarse classification of all observed expression curves. In order to get an overview on the timely orchestration of all involved biological processes, the super-clusters are roughly sorted with respect to their most active point in time, i.e. super-clusters with the highest activity at the start of the considered biological process should appear at the top of the list. If the user selects a super-cluster, e.g via a mouse-click on its curve, the according sub-clusters are shown (cf. Figure 2B). Users can also select individual clusters (cf. Figure 2B) via a mouse click or search boxes and then inspect the expression curves of all cluster members (cf. Figure 2C). The selection of any (super-)cluster directly highlights its enrichment results (cf. Figure 2D) that reveal which molecular processes are affected by the biological entities contained in the selected cluster.

### Single cell workflow

The development of modern high-throughput technologies has opened novel avenues for the genetic and molecular characterization of large sets of single cells. For example, single-cell RNA sequencing (scRNA-seq) allows to measure gene expression profiles for thousands of cells in parallel. GeneTrail’s single cell workflow was designed to explore scRNA-seq data sets in order to (i) identify for each cell the active biological processes and subsequently, based on these results, to (ii) characterize functional differences between cells types, subtypes, cell clusters etc.

Users can upload scRNA-seq data in form of a white-space separated matrix and, additionally, an annotation file containing supplemental information, like tissue and cell type, for each cell. For raw counts, GeneTrail provides several processing steps. First a user can select from a variety of filtering methods to remove artifacts like doublets or empty droplets/wells. Then a normalization is performed. A description of all processing steps can be found in Supplement S5.

For the visualization of the data set, GeneTrail processes the gene expression matrix and the annotation file to create several standard 2D representations. In particular, our web service applies Monocle 3 (24) and Seurat3 (25) to carry out dimensionality reduction (PCA, t-SNE, or UMAP), clustering, and pseudotime analysis.

In the next step, the data of each cell is processed individually. For each cell, GeneTrail carries out an over-representation analysis to identify biological processes that are (in)active in the cell.

In the last step, the enrichment results of all cells are integrated with the uploaded annotations and calculated clusters to characterize the different cell groups of interest. For each group and each pathway, GeneTrail tests if this pathway is predominantly (in)active in the cells of this group compared to all others groups. To this end, we are using a χ^2^-Test, i.e. a 2 × 2 contingency table filled with the numbers of cells in the considered group and the other groups that are (not) enriched with respect to the pathway under investigation (cf. Supplement S5).

**Figure 3.**
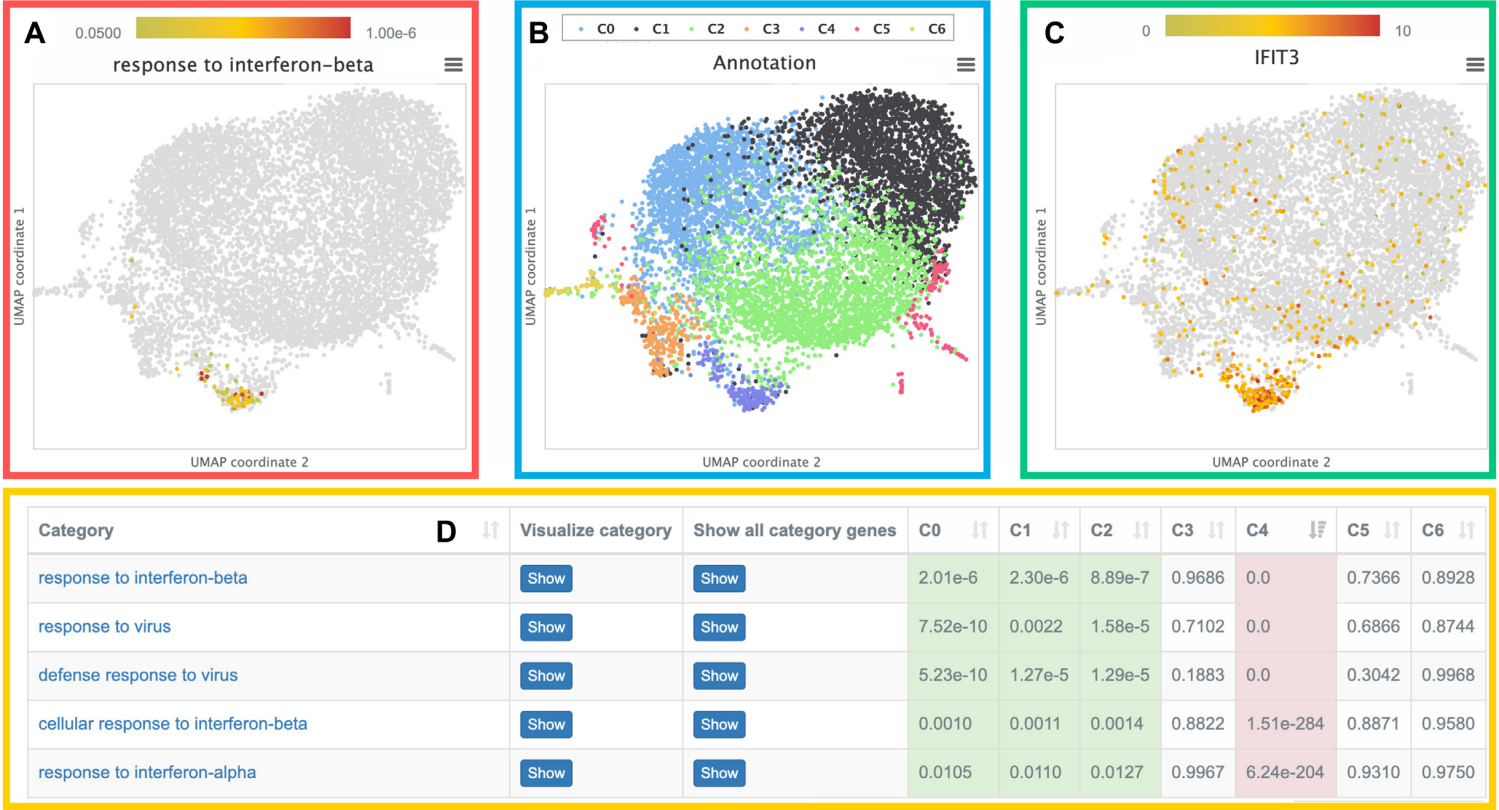
Screenshot of UMAP visualizations for single cell results of microglia cells. The different plots provide information on (**A**) the activity (*P*-values) of the biological category ‘response to interferon-beta’ in each cell, (**B**) cell clusters generated with Seurat3, (**C**) expression values of the selected gene IFIT3 and (**D**) the enrichment results (χ^2^-test) for the considered Seurat3 clustering.

All results are then presented in an interactive visualization that enables the user to study and compare expression patterns and enriched biological processes of individual cells, specific cell clusters, or predefined annotations. A screenshot of this visualization is shown in Figure 3. At the bottom, the enrichment results for the different cell groups or clusters are shown. Here, users can select interesting pathways or genes. These are then highlighted in the 2D representations at the top (cf. Figures 3A and C). The plot on the left side shows the activity of a selected pathway for each individual cell, the plot on the right side the expression of a selected gene. The plot in the middle shows the corresponding groups of cells that represent either the selected user annotation or the calculated clusters.

## TIME-SERIES ANALYSIS OF ACTIVATED T-CELLS

T cells are lymphocytes that are involved in the adaptive immune response. CD4+ cells are T cells that recruit and regulate the activity of other immune cells. Here, we consider time-resolved gene expression data of human CD4+ T cells (GSE136625) that were in vitro activated by a T cell activation kit. The data set comprises 13 expression profiles that were measured after initial T cell activation at 2 h intervals over a 24-hour period. A summary of the preprocessing and normalization of the data as well as the complete set of used parameters can be found in Supplement S6.

The major goal of our study is to deepen our knowledge of the chronological regulation of the pathways and processes induced by the T cell activation, their orchestration, and the involved regulators. Due to space constraints, we will discuss only some well-known facts that are central to T cell activation. The complete results of this analysis will be summarized in another paper. After the upload of the corresponding normalized gene expression matrix, we selected hierarchical clustering with complete linkage and Euclidean distance (of the gradients between all time points) and started the GeneTrail analysis (cf. Supplement S4). After the calculation of all gene clusters, super-clusters, and the corresponding enrichments, GeneTrail presents a summary of the results starting with the obtained 21 super-clusters. These are roughly sorted with respect to the time points of maximal activity. Hence, the first clusters should represent the processes that were immediately induced after the T cell activation. For the sake of brevity, we discuss only two of the 21 resulting super-clusters (cf. Figure 2A). The first super-cluster (SC1) contains genes with a rapid increase in expression immediately (2 hours) after T cell activation. The majority of genes (IFNG, TNF, CSF2, IL2) in SC1 belong to the products of the KEGG ‘T cell receptor signaling pathway’ (cf. KEGG - hsa04660 (18)). In fact, most of the associated biological processes are related to an early immune response. We detected an enrichment of categories related to T cell costimulation, T cell differentiation, T cell activation, and different T cell activation hallmarks (26): proliferation, cytokine signaling, and metabolic processes.

Based on these results, we can now start studying the regulation and the chronological order of downstream processes. For example, the increased expression of IFNG, induced by the TCR signaling, activates in turn the type-II interferon and the Jak-STAT signaling pathways that cause an increased production of type-I interferons. Many of these genes (23 type I and 8 type II) can be found in super-cluster SC16, whose average expression curve shows a delayed increase in expression starting after around 4 h, with a peak after 12 h. The comparison of the expression curves of the central regulators (IRF9, STAT1, STAT2) of type-I/type-II interferons with the profile of SC16 confirms this chronological sequence. Expectedly, the enriched biological categories of SC16 involve type-I/type-II interferon signaling pathways and related immune pathways, including response to external stimuli (dsRNA, virus, organic compounds etc.), natural killer cell activation, RIG-I-like receptor signaling, Toll-like receptor signaling etc.

The above discussion indicates how GeneTrail can be used to identify gene clusters, their functionality, and the chronological orchestration of the involved biological processes. Additionally, for the identification of the key regulators of each cluster, users can apply RegulatorTrail (21) that offers a variety of approaches for this task.

## SINGLE CELL ANALYSIS OF MICROGLIA CELLS

Microglia cells are a type of myeloid cells, related to macrophages, that are involved in the innate and adaptive immune defense of the central nervous system. Amongst others, they play a key role in the control of infections and the removal of necrotic neurons. An increased activation of microglia activity has also been found in several neurodegenerative disorders (27).

Here, we investigate a single cell data set of mouse microglia cells from different brain tissues (cerebellum, cortex, hippocampus and striatum). This data set is part of a comprehensive single cell transcriptome atlas that was designed to study aging processes in a large variety of mouse tissues and organs (Tabula Muris Senis Project, GSE132042, https://doi.org/10.1101/661728). The major goal of this project is to identify hallmarks of aging by comparing cells from different mouse organs, tissues, and three different age groups (3, 18 and 24 months) and by searching for features common to old cells and tissues (process and gene activities) that distinguish them from younger ones. Due to space constraints, we consider only the microglia cell data that contains 8330 gene expression profiles. Amongst others, we applied GeneTrail to study molecular processes that are characteristic for the older microglia cells (24 months). Additionally, we also examined specific cell clusters calculated using Seurat3 (cf. Figure 3B).

Older microglia cells show, amongst others, an enrichment of categories that are related to RNA processing, peptide biosynthesis, translation, and ATP synthesis. Moreover, we observe a depletion of many biological processes that belong to autophagy and regulation of autophagy. Additionally, we see a reduced expression of many heat shock proteins leading to a depletion of categories associated with response to heat, protein folding, and a reduced activity of the ‘transforming growth factor beta receptor signaling pathway’.

We have also analyzed the calculated Seurat3 clusters and discuss here only the results for cluster C1 (colored in black) and C4 (colored in lavender). For cluster C1, an enrichment of biological categories involved in development, differentiation, and morphogenesis can be observed. This indicates that these cells might be in an earlier developmental state. In fact, the uploaded age annotation confirms that the cells of this cluster predominantly belong to the 3 month cell group. The enrichment analysis for cluster C4 reveals an increased activity of processes related to a defense response against a viral infection including interferon signaling and T cell as well as natural killer cell mediated cytotoxicity (cf. Figure 3). The immune response in these cells could either be caused by a viral infection, damaged neurons, or even neurodegeneration.

The above results demonstrate how GeneTrail can be applied to study biological processes that are characteristic for specific cells or clusters. With this functionality, it provides a valuable tool for the elucidation of deregulated molecular mechanisms or even the classification of specific cells.

## DISCUSSION

Since the initial release of GeneTrail, high-throughput technologies have made a tremendous progress. They are now routinely applied in many research projects and even in clinical applications, and they usually generate noisy and extremely voluminous data sets. In order to cope with this progress, we created GeneTrail 3, a major extension of our web service.

With the new version, we provide a powerful toolbox for the elucidation of molecular mechanisms and, especially, for the identification of deregulated biological processes. It offers a large set of enrichment and network analysis algorithms that can be used to explore a comprehensive collection of biological categories for the most popular model organisms. Moreover, we have developed new powerful workflows for the analysis of epigenetic marks, time series experiments, and single cell data. For all workflows, interactive visualizations that assist users in the analysis and interpretation of the results have been created.

Due to space constraints, the presented use cases display only a small portion of the overall functionality of the new workflows and of the whole capabilities of our web service.

In summary, GeneTrail 3 offers a substantial upgrade compared to its previous versions, with several new and unique features that are of broad interest to the scientific community.

## Supplementary Material

gkaa306_Supplemental_File
