# Supplementary material for: GeneTrail 3: advanced high-throughput enrichment analysis
Source: Nucleic Acids Res. 2020 May 7;48(W1):W515–20. doi: 10.1093/nar/gkaa306 (PMC7319559; doi:10.1093/nar/gkaa306)
Supplement: gkaa306_Supplemental_File [file gkaa306_supplemental_file.pdf]

# - Supplement S1 -
GeneTrail 3: Comparison to similar tools

Method name	Supported Omics Types	Input	Objective	Algorithms/ Functionality	Supported Organisms	Accessibility	Specialized omics workflows
DAVID (1)	- genomics<br>- transcriptomics<br>- proteomics	- gene list	Analyzing large gene lists from high-throughput experiments to identify biological processes associated with investigated conditions	- ORA (Fisher's exact test)<br>- clustering of enrichment results and visualization of clustering	- Homo sapiens<br>- Mus musculus<br>- Rattus norvegicus<br>- Drosophila melanogaster	- web interface<br>- API<br>- download (EASE)<br>- R package (RDAVIDwebservice)	
Enrichr (2)	- genomics<br>- transcriptomics<br>- proteomics<br>- metabolomics<br>- epigenomics	- gene list<br>- BED files	Identifying enriched biological processses in gene lists from high-throughput experiments	- ORA (Fisher's exact test, rank score (modified Fisher's exact test), fuzzy set enrichment analysis)<br>- visualization of enrichment results (bar chart, table, grid, network)	- Homo sapiens<br>- Mus musculus<br>- Saccharomyces cerevisiae<br>- Caenorhabditis elegans<br>- Danio rerio<br>- Drosophila melanogaster	- web interface<br>- API<br>- mobile phone application	
GSEA-P (3)	-transcriptomics	- expression matrix + phenotype labels + gene set files + chip annotation files	Identifying enriched gene sets in highly correlated or anti-correlated parts of a sorted list	- GSEA (weighted Kolmogorov-Smirnov statistic)<br>- visualization of enrichment (enrichment plots)<br>- clustering of enrichment results	- Homo sapiens<br>- Mus musculus<br>- Rattus norvegicus	- download	

WebGestalt (4)	- genomics<br>- proteomics<br>- transcriptomics	-gene list	Analyzing outputs from high-throughput experiments with various enrichment based approaches and generating multiple visualizations of them	- ORA (Fisher's exact test), GSEA (weighted Kolmogorov Smirnov statistic), topology based analysis<br>- various graphical representations of enrichment results (bar chart, volcano plot, directed acyclic graph enrichment plot, pathway representation with WikiPathways)	- Arabidopsis thaliana<br>- Bos taurus<br>- Gallus gallus<br>- Sus scrofa<br>- Homo sapiens<br>- Mus musculus<br>- Rattus norvegicus<br>- Canis lupus familiaris<br>- Drosophila melanogaster<br>- Danio regio<br>- Caenorhabditis elegans<br>- Saccharomyces cerevisiae<br>- other organisms possible if user uploads gene set	- web interface<br>- API<br>- R package (WebGestaltR)	- phosphosite analysis
DIANA-miRPath (5)	- miRNomics	- miRNA list	Enrichment analysis for miRNAs	- ORA (Fisher's exact test, adjusted Fisher's exact test (EASE score), empirical sampling approach)<br>- visualization of enrichment results (table, pathway diagrams, clustering visualization)	- Homo sapiens<br>- Mus musculus<br>- Rattus norvegicus<br>- Drosophila melanogaster<br>- Danio rerio<br>- Gallus gallus<br>- Caenorhabditis elegans	- web interface	

miEAA (6)	- miRNomics	- miRNA list	Enrichment analysis for miRNAs	- ORA (Fisher's exact test)<br>- GSEA (Kolmogorov Smirnov statistic)	- Homo sapiens	- web interface	
GSEA-SNP (7)	- genomics	- SNP list	Analyzing SNPs from GWAS studies with enrichment analysis	- specialized GSEA for SNPS (Cochrane Armitage-trend test statistic)	- Homo sapiens	- R package	
i-GSEA4GWAS (8)	- genomics	- SNP or gene list (with scores)	Analyzing SNPs from GWAS studies with pathway based enrichment analysis	- specialized GSEA for SNPs (adapted Kolmogorov Smirnov statistic)<br>- functional SNP annotation analysis	- Homo sapiens	- web interface	
LOLAweb (9)	- epigenomics<br>- genomics	- BED file	Enrichment analysis for genomic regions	- ORA (Fisher's exact test)<br>- visualization of spread of query regions across chromosomes, distance from specific genomic regions	- Homo sapiens<br>- Mus musculus<br>- other organisms possible through use of custom databases	- web interface<br>- download (docker container)<br>- R package (LOLA)	

iPEAP (10)	- transcriptomics<br>- proteomics<br>- metabolomics<br>- genomics	- identifier (e.g. gene) list	Analyzing single omics data sets as well as integrative omics data sets with pathway enrichment methods	- ORA(Chi square, Fisher's exact test, binomial test), GSEA(weighted Kolmogorov Smirnov statistic), topology based enrichment (SPIA), specialized enrichment analysis for specific omics types<br>- enrichment result visualization (table)	- Homo sapiens	- download	
PaintOmics 3 (11)	- genomics<br>- transcriptomics<br>- proteomics<br>- metabolomics<br>- miRNomics<br>- region-based omics<br>- regulatory omics	- two files per omics type: measurement file + list with relevant features	Simultaneously visualizing multiple omics data by mapping them to KEGG pathways	- ORA (Fisher's exact test, Fisher combined probability test)<br>- interactive KEGG pathway based visualizations<br>- pathways interaction network<br>- enrichment resullt visualization	- all KEGG organisms	- web interface	
RAMONA (12)	- genomics<br>- transcriptomics<br>- proteomics<br>- miRNomics<br>- epigenomics	- gene list	Simultaneous analysis of various omics types using a Bayesian enrichment analysis method	- Bayesian network based approach for single omics types, Bayesian network based approach for simultaneous analysis of multiple omics types, Fisher's exact test<br>- visualization of enrichment results with bar plots, volcano plots	- Homo sapiens<br>- Mus musculus<br>- Rattus norvegicus<br>- Saccharomyces cerevisiae	- web interface<br>- partial download (MONA - does not offer full functionality)	

GeneTrail 3	- genomics<br>- transcriptomics<br>- proteomics<br>- miRNomics<br>- epigenomics	- identifier (e.g. gene) list<br>- score list<br>- measurement matrix (e.g, expression matrix): can be transformed to identifier list or score list via identifier level statistics<br>- various other input formats for specialized workflows, e.g. BED files or IDAT files (epigenomics), matrix (time series), matrix + metadata annotation (single cell)	Identification, analysis and (interactive) visualization of deregulated pathways using enrichment analysis based methods	- Identifier level statistics: fold change, z-score, signal-to-noise ratio, Pearson correlation, Spearman correlation, F-test, t-test, Wilcoxon rank-sum test, Wilcoxon matched-pairs signed ranks test<br>- ORA (Hypergeometric Test, Fisher's exact test), GSEA ((weighted) Kolmogorov Smirnov statistic), others<br>- visualization of enrichment results (interactive tables)<br>- specialized visualization for epigenomics, single cell, time series and regulatory genomics (clustering, dimension reduction, interactive figures)<br>- seamless integration with tools for network analysis (NetworkTrail, FiDePa), drug target analysis (DrugTargetInspector, ClinOmicsTrail) and regulatory genomics (RegulatorTrail)	- Arabidopsis thaliana<br>- Bos taurus<br>- Gallus gallus<br>- Sus scrofa<br>- Homo sapiens<br>- Mus musculus<br>- Rattus norvegicus<br>- Canis lupus familiaris<br>- Drosophila melanogaster<br>- Danio rerio<br>- Caenorhabdit is elegans<br>- Pan troglodytes	- web interface<br>- API	- epigenomics<br>- single cell<br>- time series<br>- regulatory genomics

References

[1] Huang, D. W., Sherman, B. T., and Lempicki, R. A. (2009) Systematic and integrative analysis of large gene lists using DAVID bioinformatics resources. *Nature protocols*, **4(1)**, 44–57.

[2] Kuleshov, M. V., Jones, M. R., Rouillard, A. D., Fernandez, N. F., Duan, Q., Wang, Z., Koplev, S., Jenkins, S. L., Jagodnik, K. M., Lachmann, A., McDermott, M. G., Monteiro, C. D., Gundersen, G.W. and McDermott, M. G. (2016) Enrichr: a comprehensive gene set enrichment analysis web server 2016 update. *Nucleic acids research*, **44(W1)**, W90–W97.

[3] Subramanian, A., Kuehn, H., Gould, J., Tamayo, P., and Mesirov, J. P. (2007) GSEA-P: a desktop application for Gene Set Enrichment Analysis. *Bioinformatics*, **23(23)**, 3251–3253.

[4] Liao, Y., Wang, J., Jaehnig, E. J., Shi, Z., and Zhang, B. (2019) WebGestalt 2019: gene set analysis toolkit with revamped UIs and APIs, *Nucleic Acids Research*, **47(W1)**, W199–W205.

[5] Vlachos, I. S., Zagganas, K., Paraskevopoulou, M. D., Georgakilas, G., Karagkouni, D., Vergoulis, T., Dalamagas, T. and Hatzigeorgiou, A. G. (2015). DIANA-miRPath v3. 0: deciphering microRNA function with experimental support. *Nucleic acids research*, **43(W1)**, W460–W466.

[6] Backes, C., Khaleeq, Q. T., Meese, E., and Keller, A. (2016) miEAA: microRNA enrichment analysis and annotation. *Nucleic acids research*, **44(W1)**, W110–W116.

[7] Holden, M., Deng, S., Wojnowski, L., and Kulle, B. (2008) GSEA-SNP: applying gene set enrichment analysis to SNP data from genome-wide association studies. *Bioinformatics*, **24(23)**, 2784–2785.

[8] Zhang, K., Chang, S., Guo, L., and Wang, J. (2015) I-GSEA4GWAS v2: a web server for functional analysis of SNPs in trait-associated pathways identified from genome-wide association study. *Protein & cell*, **6(3)**, 221–224.

[9] Nagraj, V. P., Magee, N. E., and Sheffield, N. C. (2018) LOLAweb: a containerized web server for interactive genomic locus overlap enrichment analysis. *Nucleic acids research*, **46(W1)**, W194-W199.

[10] Sun, H., Wang, H., Zhu, R., Tang, K., Gong, Q., Cui, J., Cao, Z., and Liu, Q. (2013) iPEAP: integrating multiple omics and genetic data for pathway enrichment analysis. *Bioinformatics*, **30(5)**, 737–739.

[11] Hernández-de-Diego, R., Tarazona, S., Martínez-Mira, C., Balzano-Nogueira, L., Furió-Tarí, P., Pappas Jr, G. J., and Conesa, A. (2018) PaintOmics 3: a web resource for the pathway analysis and visualization of multi-omics data. *Nucleic acids research*, **46(W1)**, W503–W509.

[12] Sass, S., Buettner, F., Mueller, N. S. and Theis, F. J. (2015) RAMONA: a Web application for gene set analysis on multilevel omics data. *Bioinformatics*, **31(1)**, 128–130.

# - Supplement S2 -
GeneTrail 3: Data Provenance

1 Supported organisms

Latin name	Common name	Taxon ID
Homo sapiens	Modern human	9606
Mus musculus	House mouse	10090
Rattus norvegicus	Brown rat	10116
Arabidopsis thaliana	Thale cress	3702
Danio rerio	Zebrafish	7955
Drosophila melanogaster	Fruit fly	7227
Caenorhabditis elegans	Roundworm	6239
Bos taurus	Cow (cattle)	9913
Canis familiaris	Domestic dog	9615
Gallus gallus	Chicken	9031
Pan troglodytes	Common chimpanzee	9598
Sus scrofa	Domestic pig	9823

Table 1: Supported organisms.

2 Supported identifier types

Identifier	Example
Entrez gene	602
Official gene symbol	BCL3
Gene alias	BCL4
Ensembl gene	ENSG00000012048
Ensembl transcript	ENST00000264227
RefSeq gene	NC_000019
RefSeq transcript	XM_005259129
Vega gene	OTTHUMG00000151517
Vega RNA	OTTHUMT00000322976
NCBI GI gene	148151281
NCBI GI RNA	221041195
UniGene	Hs.31210

Table 2: Gene identifier.

Identifier	Example
Ensembl protein	ENSP00000164227
Vega protein	OTTHUMP00000200151
NCBI GI protein	578822694
UniprotKB AC/ID	P20749
UniRef100	UniRef100_P20749
UniRef90	UniRef90_P20749
UniRef50	UniRef50_P20749
UniParc	UPI0000D4AF29

Table 3: Protein identifier.

Identifier	Example
miRBase (V14 - V21)	hsa-miR-15b-3p
miRBase Accession	MIMAT0004585

Table 4: miRNA identifier.

Identifier	Example
dbSNP	rs34039386

Table 5: SNP identifier.

3 Databases

Omics	Database
mRNA + Protein	ConsensusPathDB [16], CORUM [1], EpiFactors [2], GO [3], HumanCyc [4], HumanProteinAtlas [5], KEGG [6], miRTarBase [7], MSigDB [8], PANTHER [9], Pfam [10], PharmGKB [11], Reactome [12], RefSeq [13], TRANSFAC [15], WikiPathways [14]
miRNA	GO [3], miRTarBase [7], mirPathDB [17]
SNPs	GWAS Catalog [18], PheWAS Catalog [19]

Table 6: Databases integrated into GeneTrail2.

References

[1] Giurgiu, M., Reinhard, J., Brauner, B., Dunger-Kaltenbach, I., Fobo, G., Frishman, G., et al. (2019) CORUM: the comprehensive resource of mammalian protein complexes—2019. *Nucleic acids research*, **47(D1)**, D559-D563.

[2] Kim, J. H. (2019). Epigenome Database and Analysis Tools. *In Genome Data Analysis*, 339-352.

[3] Gene Ontology Consortium. (2019). The gene ontology resource: 20 years and still GOing strong. *Nucleic acids research*, **47(D1)**, D330-D338.

[4] Caspi, R., Billington, R., Foerster, H., Fulcher, C. A., Keseler, I., Kothari, A., et al. (2016) BioCyc: online resource for genome and metabolic pathway analysis. *The FASEB Journal*, $\mathbf{30(1}_supplement), lb192 - lb192$.

[5] Thul, P. J., and Lindskog, C. (2018) The human protein atlas: A spatial map of the human proteome. *Protein Science*, **27(1)**, 233-244.

[6] Kanehisa, M., Furumichi, M., Tanabe, M., Sato, Y., and Morishima, K. (2017) KEGG: new perspectives on genomes, pathways, diseases and drugs. *Nucleic acids research*, **45(D1)**, D353–D361.

[7] Chou, C. H., Shrestha, S., Yang, C. D., Chang, N. W., Lin, Y. L., Liao, K. W., et al. (2018) miRTarBase update 2018: a resource for experimentally validated microRNA-target interactions. *Nucleic acids research*, **46(D1)**, D296-D302.

[8] Liberzon, A., Subramanian, A., Pinchback, R., Thorvaldsdóttir, H., Tamayo, P., and Mesirov, J. P. (2011). Molecular signatures database (MSigDB) 3.0. *Bioinformatics*, **27(12)**, 1739-1740.

[9] Mi, H., Huang, X., Muruganujan, A., Tang, H., Mills, C., Kang, D., and Thomas, P. D. (2017). PANTHER version 11: expanded annotation data from Gene Ontology and Reactome pathways, and data analysis tool enhancements. *Nucleic acids research*, **45(D1)**, D183-D189.

[10] El-Gebali, S., Mistry, J., Bateman, A., Eddy, S. R., Luciani, A., Potter, S. C., et al. (2019) The Pfam protein families database in 2019. *Nucleic acids research*, **47(D1)**, D427-D432.

[11] Barbarino, J. M., Whirl-Carrillo, M., Altman, R. B., and Klein, T. E. (2018) PharmGKB: a worldwide resource for pharmacogenomic information. *Wiley Interdisciplinary Reviews: Systems Biology and Medicine*, **10(4)**, e1417.

[12] Fabregat, A., Jupe, S., Matthews, L., Sidiropoulos, K., Gillespie, M., Garapati, P., et al. (2018) The reactome pathway knowledgebase. *Nucleic acids research*, **46(D1)**, D649-D655.

[13] Haft, D. H., DiCuccio, M., Badretdin, A., Brover, V., Chetvernin, V., O'Neill, K., et al. (2018) RefSeq: an update on prokaryotic genome annotation and curation. *Nucleic acids research*, **46(D1)**, D851-D860.

[14] Kelder, T., Van Iersel, M. P., Hanspers, K., Kutmon, M., Conklin, B. R., Evelo, C. T., and Pico, A. R. (2012) WikiPathways: building research communities on biological pathways. *Nucleic acids research*, **40(D1)**, D1301-D1307.

[15] Kel, A., and Tatarinova, T. (2017). TRANSFAC-ing the rice genome. *In Moscow Conference on Computational Molecular Biology*, **MCCMB 2017**, 32.

[16] Herwig, R., Hardt, C., Lienhard, M., and Kamburov, A. (2016). Analyzing and interpreting genome data at the network level with ConsensusPathDB. *Nature protocols*, **11(10)**, 1889.

[17] Kehl, T., Kern, F., Backes, C., Fehlmann, T., Stöckel, D., Meese, E., ... and Keller, A. (2020). miRPathDB 2.0: a novel release of the miRNA Pathway Dictionary Database. *Nucleic acids research*, **48(D1)**, D142-D147.

[18] MacArthur, J., Bowler, E., Cerezo, M., Gil, L., Hall, P., Hastings, E., et al. (2017) The new NHGRI-EBI Catalog of published genome-wide association studies (GWAS Catalog). *Nucleic acids research*, **45(D1)**, D896-D901.

[19] Denny, J. C., Bastarache, L., Ritchie, M. D., Carroll, R. J., Zink, R., Mosley, J. D., et al. (2013). Systematic comparison of phenome-wide association study of electronic medical record data and genome-wide association study data. *Nature biotechnology*, **31(12)**, 1102.

# - Supplement S3 -
GeneTrail 3: Epigenomics Workflow

1 Motivation

The GeneTrail epigenomics workflow provides functionality to analyze histone marks, DNA methylation patterns, and open-chromatin regions of two or more sample groups. The epigenetic marks are used to identify genes that transition between certain chromatin states in the different groups. The gene sets of the different transition groups are then used to analyze affected signaling pathways. To this end, several analysis steps are carried out: First, our web service identifies, which epigenetic modifications are present in the promoter, enhancer and gene body regions of each transcript. This information then defines a combined chromatin state for all regulatory and coding regions that influence this transcript, i.e. active, poised, repressed, or no information. In the following sections, we call this the chromatin state of a transcript. The transcript information is then used to define the chromatin state of each gene. In the last step, The epigenetic marks are used to divide the genes into transition groups, e.g. genes that transition from a certain chromatin state in one sample to another state in a second sample. For all resulting transition groups, enrichment analyses are then carried out to identify associated pathways. An overview of the workflow is shown in Figure 1.

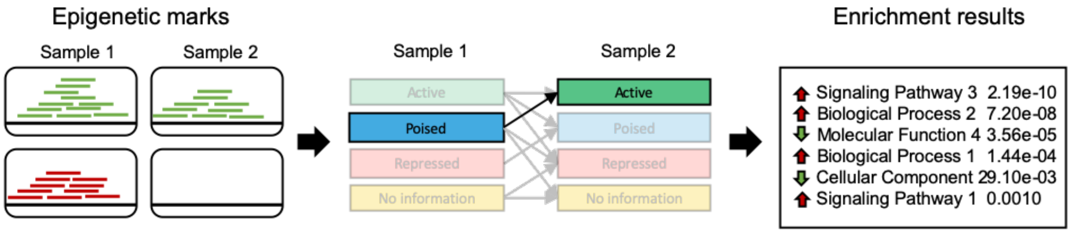

Figure 1: Overview of the epigenomics workflow.

2 Input data

The input for the epigenomics workflow are genomic positions affected by a certain epigenetic mark. Currently, we support open chromatin regions, histone modifications, DNA methylation patterns, and associated gene expression measurements. In the following sections, we describe the file formats that can be used to upload different data sources.

2.1 Open chromatin regions or histone modifications

Open chromatin regions or histone modifications are expected to be given as peaks in a BED file format.

2.1.1 BED format

In this format every line represents a region of interest. Each individual line contains at least three fields.

1. Chromosome
2. Start position of the region
3. End position of the region

Example:

```
chr1 180775 180925
chr1 181395 181545
chr1 273895 274045
chr1 629895 630045
chr1 633855 634005
...
```

2.2 DNA methylation patterns

DNA methylation can either be uploaded as IDAT file resulting from Illumina BeadArrays or as BED file from bisulfite sequencing experiments.

2.3 Gene expression patterns

RNA-Seq input has to be a tab-separated matrix of gene- or transcript expression values. The rows of this matrix are expected to be gene or transcript identifiers and the columns should represent measured samples. These samples can include replicates for each group. The matrix has to have row names and a header. All expression measurements have to be uploaded in a single matrix. After the matrix was successfully uploaded and parsed, a table will appear in which you can assign each measurement (column in the matrix) to a group.

2.4 Naming convention

After a file has been uploaded, it must be assigned to the associated epigenetic mark. In case the name of the uploaded file contains the mark this assignment is done automatically.

2.5 Upload

All files can either be uploaded individually or in form of a ZIP archive. After the files have been uploaded, they have to be assigned to the investigated sample groups.

3 Identifying epigenetic modifications in promoter, enhancer, and gene body regions of transcripts

We define a promoter of a transcript as window around the transcription start site (TSS). This window size is a parameter and can be 1000 base pairs (also written as 1kb), 2kb, or 5kb, resulting in a total promoter size of 2001, 4001 and 10001 bp, respectively, for each transcript. The gene body is defined as the union of the genomic positions of all exons. Enhancer regions are taken from the database GeneHancer [1]. This database comprises regions of possible enhancers and their possible gene targets for humans. The genomic positions for the TSS and exons are taken from GENCODE [2]. A promoter, enhancer, or gene body region is stated to be affected by an epigenetic mark, if at least one bp of the region is affected by the measured mark. For histone modifications and open-chromatin regions, we use bedtools [3] to test for an intersection of the genomic positions. For DNA methylation, we use RnBeads [4] to calculate a beta-value for each region that states the degree of DNA methylation in this region. We choose 0.8 as beta-value cutoff. As a result, each transcript is assigned a set of epigenetic marks for its regulatory and gene body regions.

4 Predicting a combined chromatin state of promoter, enhancer, and gene body regions of a transcript

An epigenetic mark can have a positive or negative effect on transcription, depending on its location. The tri-methylation of lysine 36 in histone 3 (in short H3K36me3) for example can positively affect transcription if it is found in the gene body [5, 6]. Another example is DNA methylation that can have a negative effect on transcription if found in promoter regions [7]. In our analysis, the epigenetic modification pattern, affecting the regulatory and gene body regions of a transcript, is analyzed based on this a priori knowledge gathered from the specialized databases HIstome [8] and HHMD (Human Histone Modification Databse) [9], as well as from a manual literature search to complement this knowledge. Based on decision rules extrated from this knowlege base, we can assign a combined chromatin state to those regions, which is either 'active',

'poised', or 'repressed'. If no epigenetic marks are present in the promotor, enhancer, or gene body regions of a transcript, the chromatin state is set to 'no information'.

5 Predicting the chromatin state of a gene

If a gene codes for several transcripts, it might occur that the chromatin states for the regulatory and gene body regions of those transcripts are assigned to different values. In that case, we assume that the function of these transcripts are redundant and that any transcript can fulfill the function of the gene. Therefore, we assign a gene to the most active chromatin state of its transcripts. Hence, if at least one transcripts is active, the gene is assigned to be active. If this is not the case and if at least one transcripts is poised, the gene is assigned to be poised. If all transcripts are repressed, the gene is also assigned to be repressed. In the case that no transcript contains any epigenetic mark, the gene is assigned to have no information.

6 Performing enrichment analyses

In the last step of the workflow, the different uploaded sample groups are compared using enrichment analyses. For each pair of groups, we define 16 sets of genes, one for each chromatin state pair. Thereby, chromatin state pairs represent transitions between chromatin states of two groups. A gene is assigned to a chromatin state pair (e.g., active - repressed) if it is assigned to the first chromatin state in the first group (in the example active) and to the second chromatin state in the second group (in the example repressed). For these 16 sets of genes, we perform an Over-Representation Analysis (ORA).

6.1 Over-representation analysis

Let us assume that we have a biological category (signaling pathway or biological process) that has k entries in our test set, which consists of n entries, and l entries in the reference (all investigated genes), which consists of m entries. We can then use one of the following statistical tests to check if the test set has more entries in our category then expected by chance.

6.1.1 Hypergeometric test

For this analysis, all elements of test set are always part of the reference. For this purpose, the hypergeometric test can be applied to compute a p-value for the analyzed category:

$$P(K >= k) = \sum_{i=max(n+l-m,0)}^{k} \frac{lim-ln-i}{mn}$$

6.1.2 Multiple testing correction

Since for each test set in our analysis multiple biological categories are tested simultaneously, we need to adjust the resulting p-values in order the account for the multiple testing problem. For this purpose GeneTrail provides a variety of methods (cf. [10]).

References

[1] Fishilevich, S., Nudel, R., Rappaport, N., Hadar, R., Plaschkes, I., Iny Stein, T., ... and Lancet, D. (2017). GeneHancer: genome-wide integration of enhancers and target genes in GeneCards. Database, 2017.

[2] Frankish, A., Diekhans, M., Ferreira, A. M., Johnson, R., Jungreis, I., Loveland, J., ... and Barnes, I. (2019). GENCODE reference annotation for the human and mouse genomes. Nucleic acids research, 47(D1), D766-D773.

[3] Quinlan, A. R., and Hall, I. M. (2010). BEDTools: a flexible suite of utilities for comparing genomic features. Bioinformatics, 26(6), 841-842.

[4] Müller, F., Scherer, M., Assenov, Y., Lutsik, P., Walter, J., Lengauer, T., and Bock, C. (2019). RnBeads 2.0: comprehensive analysis of DNA methylation data. Genome biology, 20(1), 55.

[5] Venkatesh, S., Li, H., Gogol, M. M., and Workman, J. L. (2016). Selective suppression of antisense transcription by Set2-mediated H3K36 methylation. Nature communications, 7(1), 1-14.

[6] Li, B., Carey, M., and Workman, J. L. (2007). The role of chromatin during transcription. Cell, 128(4), 707-719.

[7] Jones, P. A. (2012). Functions of DNA methylation: islands, start sites, gene bodies and beyond. Nature Reviews Genetics, 13(7), 484-492.

[8] Khare, S. P., Habib, F., Sharma, R., Gadewal, N., Gupta, S., and Galande, S. (2012). HIstome—a relational knowledgebase of human histone proteins and histone modifying enzymes. Nucleic acids research, 40(D1), D337-D342.

[9] Zhang, Y., Lv, J., Liu, H., Zhu, J., Su, J., Wu, Q., ... and Li, X. (2010). HHMD: the human histone modification database. Nucleic acids research, 38(suppl_1), D149-D154.

[10] Stöckel, D., Kehl, T., Trampert, P., Schneider, L., Backes, C., Ludwig, N., ... and Meese, E. (2016). Multi-omics enrichment analysis using the GeneTrail2 web service. Bioinformatics, 32(10), 1502-1508.

# - Supplement S4 -
GeneTrail 3: Time Series Workflow

1 Motivation

The GeneTrail time series workflow provides functionality for the analysis of time resolved gene, protein, or miRNA expression data sets. In order to process these data sets, several analysis steps are conducted: our web service first uses a two-stage clustering approach to group biological entities with very similar expression patterns over time. For the resulting clusters, enrichment analyses are carried out to identify associated pathways. An overview of the workflow is shown in Figure 1.

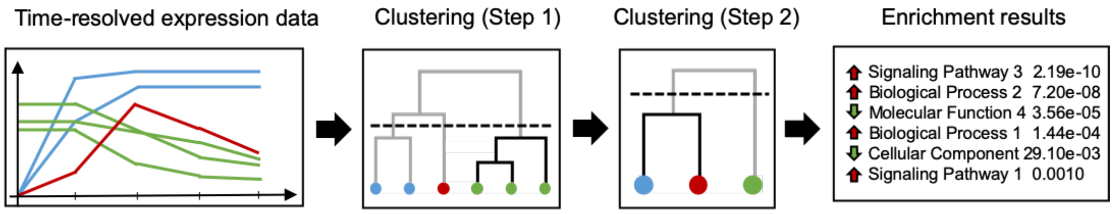

Figure 1: Overview of the time series workflow.

2 Input data

The input for a time series analysis are gene expression values obtained from microarrays, RNA-seq experiments or mass-spectrometry runs. The measurements can be uploaded as a plain text, tab-separated matrix where columns represent specific time points and each row represents the expression measurements for a particular gene at those time points.

Example:

```
TimePoint1 TimePoint2 TimePoint3
GeneA 0.1 4.3 2.3
GeneB 3.2 1.2 1.1
GeneC 2.7 9.1 0.3
...
```

In general we recommend to upload already normalized and logarithmized data. This gives users the complete control over quality control, batch effect removal, and normalization. However, for RNA-Seq data GeneTrail providesa variety of normalization methods: log(TMM+1) [2], log(GeTMM+1) [3], log(TPM+1) [1], log(CPM+1).

3 Clustering

In order to group genes, proteins or miRNA with similar expression patterns, we carry out two clustering steps. First, a strict clustering is performed that generates small groups with a high similarity between all members. The second clustering then combines similar clusters generated in the first step to "super-clusters". These super-clusters give a general overview of the data. However, they can be further refined to investigate the contained subclusters and finally also the individual genes.
For each stage of our clustering approach, we need to carry out four steps: (1) filtering the gene expression data , (2) calculating the distance between all gene pairs, (2) hierachical clustering, and (3) cutting the dendrogram to obtain clusters.

3.1 Filtering

In a preprocessing step, we remove all genes that only show limited expression changes over time. To this end, we implemented two metrics that summarize the overall expression changes in a time series $t = \{t_1, ..., t_n\}$. The result of a metric is then compared to a user defined threshold δ to remove unwanted genes, i.e. genes with low overall expression changes with respect to the chosen metric.

3.1.1 Absolute expression difference

The first measure calculates the absolute difference between the highest and lowest expression value in t.

$$d(t) = max(t) - min(t)$$

3.1.2 Average expression change

The second method calculates the average expression difference over all consecutive time points.

$$d(t) = \frac{1}{n-1} \sum_{i=1}^{n-1} |x_i - x_{i+1}|$$

3.2 Distance measures

In order to quantify the distance of two time courses, we implemented a variety of distance measures. For the description of these measures, we consider both curves as n-dimensional vectors $p = (p_1, p_i, ..., p_n)$ and $q = (q_1, q_i, ..., q_n)$, where $i \in \{1, ..n\}$ represents time point i.

3.2.1 Distance measures for time points

In this section, we describe distance measures that calculate the distance between p and q based on their entries.

3.2.1.1 Euclidean distance

The Euclidean distance is a metric for distance between two points in Euclidean space. It is defined as

$$d(p,q) = \sqrt{\textstyle\sum_{i=1}^{n}(q_i - p_i)^2}$$

3.2.1.2 Minimized Euclidean distance

For the minimized version of the Euclidean distance, we shift one of the vectors such that the distance between the two is minimized. This means that height differences between the curves are ignored.

$$d(p,q) = \sqrt{\textstyle\sum_{i=1}^{n}(q_i - s - p_i)^2}$$

The optimal value for s can be found as follows:

$$s = \tfrac{1}{n}\textstyle\sum_{i=1}^{n}(q_i - p_i)$$

3.2.2 Distance measures for transitions between time points

In this section, we describe distance measures that consider the transitions between the time points rather than the time points themselves.

3.2.2.1 Angle distance

We define the angle distance as the sum of all angles between the transitions of consecutive time points in the time series.

$$d(p,q) = \textstyle\sum_{i=1}^{n-1}\theta((1, p_{i+1} - p_i), (1, q_{i+1} - q_i))$$

where θ is defined as the angle between the transition of two consecutive points.

3.2.2.2 Euclidean distance for gradients

This version of the Euclidean distance, calculates the distance between p and q based on the gradients of all consecutive time points.

$$d(p,q) = \sqrt{\sum_{i=1}^{n-1}((q_{i+1} - q_i) - (p_{i+1} - p_i))^2}$$

3.2.3 Association measures

In addition to the distance measures described above, we can also use a variety of association/similarity measures. Before a clustering can be applied, these values have to be transformed to distance measures.

3.2.3.1 Pearson correlation

The Pearson correlation coefficient (PCC) is a measure for linear dependence between two random variables P and Q. We assume that p and q are samples drawn from these variables.

$$r(p,q) = \frac{1}{n-1}\sum_{i=1}^{n}(\frac{p_i-\bar{p}}{s_P})(\frac{q_i-\bar{q}}{s_Q}),$$

where $\bar{p}, \bar{q}$ and s_P, s_Q are the sample means and samples variances respectively.

The Pearson correlation coefficient r ranges from -1 to 1. A value of 1 implies that the relationship between P and Q is perfectly described by a linear function, with all data points lying on a line for which both P and Q increase. A value of 1 implies that all data points lie on a line for which P increases as Q decreases. A value of 0 implies that there is no linear dependence between the variables. This means we can define the following distance measure:

$$d(p,q) = 1 - r(p,q)$$

3.2.3.2 Spearman correlation

The Spearman correlation coefficient (SCC) is a non-parametric measure for dependence between between two random variables P and Q. Here, we assume that p and q are samples drawn from these variables. The SCC assesses how well the relationship between two variables can be described using a monotonic function.

$$r(p,q) = 1 - \frac{6\sum_{i=1}^{n}(rank(p_i)-rank(q_i))^2}{n(n^2-1)}$$

The rank $rank(x_i)$ of a sample x_i is the position of that sample in the decreasingly ordered sequence of all samples.

In accordance with the PCC, we can transform the SCC into a distance measure:

$$d(p, q) = 1 - r(p, q)$$

3.3 Hierarchical clustering

For the hierarchical clustering, we rely on the *'fastcluster'* R-package (https://cran.r-project.org/web/packages/fastcluster/index.html). It provides an agglomerative clustering approach, where each observation starts in its own cluster and pairs of clusters are merged until a complete hierarchy is generated. In each step, all the clusters with the smallest distance are merged. In order to identify the minimum distance of two clusters A and B, users can choose from a variety of methods.

3.3.1 Average Linkage

$d(A, B) = \frac{1}{|A||B|} \sum_{a \in A, b \in B} d(a, b)$

3.3.2 Complete Linkage

$d(A, B) = \max_{a \in A, b \in B} \{d(a, b)\}$

3.3.3 Single Linkage

$d(A, B) = \min_{a \in A, b \in B} \{d(a, b)\}$

3.3.4 McQuitty

$d(A, B) = \frac{1}{(|A|+|B|)(|A|+|B|-1)} \sum_{a,b \in A \cup B} d(a, b)$

3.3.5 Ward's Method

$d(A, B) = \frac{d(\bar{a}, \bar{b})}{\frac{1}{|A|} + \frac{1}{|B|}}$

3.4 Final clusters

The resulting dendrogram contains the complete cluster hierarchy. In order to obtain a set of clusters that is of special interest to the user, the dendrogram has to be cut. To this end, users can define a threshold $t \in [0, 1]$ that describes the quantile of merges that should be used to generate a final result. A smaller threshold will result in a stricter clustering with more individual clusters that have a higher similarity between all members.

4 Over-representation analysis

For the resulting clusters and super-clusters, enrichment analyses are carried out to identify associated pathways. To this end, lets assume that we have a biological category (signaling pathway or biological process) that has k entries in our test set (cluster), which consists of n entries, and l entries in the reference (all investigated genes), which consists of m entries. We can then use one of the following statistical tests to check if the test set has more entries in our category then expected by chance.

4.1 Hypergeometric test

For this analysis, all elements of test set is always part of the reference. For this purpose, the hypergeometric test can be applied to compute a p-value for the analyzed category:

$$P(K >= k) = \sum_{i=max(n+l-m,0)}^{k} \frac{lim-ln-i}{mn}$$

4.2 Multiple testing correction

Since for each cluster in our analysis multiple biological categories are tested simultaneously, we need to adjust the resulting p-values in order the account for the multiple testing problem. For this purpose GeneTrail provides a variety of methods (cf. [4]).

References

[1] Wagner, G. P., Kin, K., and Lynch, V. J. (2012). Measurement of mRNA abundance using RNA-seq data: RPKM measure is inconsistent among samples. Theory in biosciences, 131(4), 281-285.

[2] Robinson, M. D., and Oshlack, A. (2010). A scaling normalization method for differential expression analysis of RNA-seq data. Genome biology, 11(3), R25.

[3] Smid, M., van den Braak, R. R. C., van de Werken, H. J., van Riet, J., van Galen, A., de Weerd, V., ... and Wilting, S. M. (2018). Gene length corrected trimmed mean of M-values (GeTMM) processing of RNA-seq data performs similarly in intersample analyses while improving intrasample comparisons. BMC bioinformatics, 19(1), 236.

[4] Stöckel, D., Kehl, T., Trampert, P., Schneider, L., Backes, C., Ludwig, N., ... and Meese, E. (2016). Multi-omics enrichment analysis using the GeneTrail2 web service. Bioinformatics, 32(10), 1502-1508.

# - Supplement S5 -
GeneTrail 3: Single Cell Workflow

1 Motivation

The GeneTrail single cell workflow provides functionality to analyze single cell RNA sequencing (scRNA-seq) data sets. Particularly, it is designed to (1) identify for each cell active biological processes, and, (2) subsequently, based on these results, to characterize functional differences between cells. In order to process scRNA-seq data sets, several analysis steps are conducted: First, we optionally normalize the user uploaded RNA-seq matrix. Then, we conduct an enrichment analysis for each single-cell to identify pathways associated with the expressed genes. Additionally, we cluster the cells and perform dimension reduction. As a last step, we group individual cells based on cell clusters or uploaded annotations, e.g. tissue or sample id, and find pathways that are associated with specific groups. An overview of the workflow is shown in Figure 1.

2 Input data

The input for our single cell workflow is a gene expression matrix obtained from a scRNA-Seq run and metadata file. Both can be uploaded as plain text files.

2.1 Expression matrix

The expression matrix is assumed to be a tab-separated matrix where columns represent specific cells and each row represents the expression measurements for a particular gene in those cells. The content of the matrix can either be read/UMI counts or normalized expression values.

Example:

```
Cell1 Cell2 Cell3
GeneA 0 4 0
GeneB 2 1 0
GeneC 2 19 5
...
```

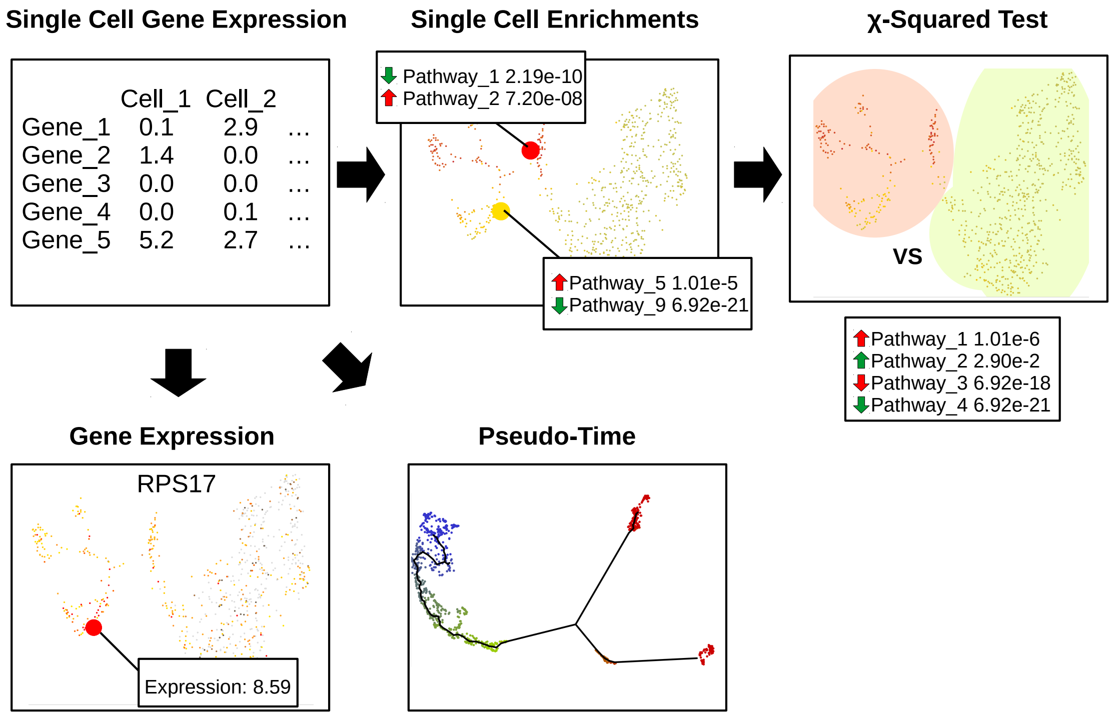

Figure 1: Overview of the single cell workflow.

2.2 Metadata

The metadata file can be uploaded as tab-separated text file in which each column provides additional meta information for the cells that should be analyzed. The cell identifier have to match with the column names of the scRNA-Seq matrix. Only cells with an entry in both, the metadata file, and the scRNA-Seq matrix, are analyzed in the subsequent workflow.

```
MetaInfo1 MetaInfo2 MetaInfo3
Cell1 age-3 batch_1 cluster_6
Cell2 age-5 batch_1 cluster_2
Cell3 age-3 batch_2 cluster_6
...
```

3 Filtering and normalization

As already mentioned, users are able to either upload preprocessed and normalized expression values or raw count data. For raw counts, GeneTrail provides several processing steps that are discussed in the following sections.

3.1 Filtering

Current scRNA-seq protocols involve steps to isolate single cells. During this process several artifacts can occur. Some of the cells might not have been separated properly, which could result in doublets, while others could potentially be damaged. GeneTrail provides several filtering procedures that help to remove these artifacts. To this end, we follow the best practices guide by Luecken and Theis [1].

3.1.1 Removing damaged cells

A low number of UMI/read counts, a low number of expressed genes and a high percentage of mitochondrial gene counts might be an indication for cells with a broken membrane, where the mRNA in the cytoplasm might have leaked out. Hence, users can select thresholds for each of those criterions to remove affected cells.

3.1.2 Removing doublets

Doublets can potentially be detected by a very high number of UMI/read counts or by a very high number of expressed genes. Here, users are again able to select thresholds in order to exclude affected cells from further analyses.

3.2 Normalization

For our analysis to work properly, we require a within-sample normalization, as we compare the expression values of different genes. This means that depending on the used scRNA-Seq protocol (e.g. for full-length sequencing protocols like SMART-seq2) a gene length normalization is required. Additionally, normalized expression values should be log transformed (log(x+1)). Currently, we offer two methods for gene-length normalization: TPM [4] and GeTMM [6], and two methods for between-sample normalization: CPM [**?**] and TMM [5]. Based on the selected protocol, we already preselect suitable default parameters. After normalization all expression values are log transformed ($log_2(x+1)$).

4 Enrichment analysis of individual cells

Next, for each single cell, an enrichment analysis is conducted. To this end, we consider only the most expressed genes of the cell. Therefore, the user can determine a threshold for filtering the genes that should be further analyzed. Alternatively, we use the x genes with the highest normalized expression values. In both cases, we generate one gene set for each single cell, independently. The gene sets are used as test sets in an Over-Representation Analysis (ORA) [2, 3]. As a reference set all protein coding genes are used.

4.1 Over-representation analysis

Let us assume that we have a biological category (signaling pathway or biological process) that has k entries in our test set, which consists of n entries, and l entries in the reference, which consists of m entries. We can then use one of the following statistical tests to check if the test set has more entries in our category then expected by chance.

4.1.1 Hypergeometric test

If all elements of the test set are also a part of the reference, the hypergeometric test is applied to compute a p-value for the analyzed category:

$$P(K >= k) = \sum_{i=max(n+l-m,0)}^{k} \frac{\binom{l}{i}\binom{m-l}{n-i}}{\binom{m}{n}}$$

4.1.2 Fisher's exact test

If the test set contains elements that are not a part of the reference, the Fisher's exact test is applied to compute a p-value for the analyzed category:

$$P(K >= k) = \sum_{i=max(l+k-m,0)}^{k} \frac{\binom{n}{i}\binom{m}{l+k-i}}{\binom{m+n}{l+k}}$$

4.1.3 Multiple testing correction

Since for each cell in our single cell analysis multiple biological categories are tested simultaneously, we need to adjust the resulting p-values in order the account for the multiple testing problem. For this purpose GeneTrail provides a variety of methods (cf. [3]).

	e	$\neg e$
i	c_{11}	c_{12}
$\neg i$	c_{21}	c_{22}

Table 1: The 2×2 contingency table used to test if pathway p is more often found to be enriched in group g compared to what is expected given all other groups. The variable e represents cells in which pathway p is enriched. The variable i represents cells that are contained in group g.

5 Group comparison of enrichment results

Along with the scRNA-seq expression matrix, a user can upload metadata for each cell (e.g. tissue of origin, sample id, or clinical information). This information can be used to define groups of special interest to the user. Additionally, we cluster the cells in our workflow independently from the given annotations using Seurat3 [7, 8]. Subsequently, the clusters can also be used to partition the cells into groups.

In order to find pathways that are associated with certain groups, we perform a χ-squared test on the previously generated enrichment results. The intuition behind the χ-squared test is to identify pathways that are more often enriched in cells from one particular group compared to all other groups. To this end, we perform one χ-squared test for each pathway and for each group.

The χ-squared test for two variables can be defined over a 2×2 contingency table. Let p be a pathway and g be a group of interest. Furthermore, let n be the total number of cells. We create a 2×2 contingency table as shown in Table 1. The columns indicate if pathway p is enriched for a cell and the rows indicate if a cell is contained in group g. The cells in the contingency table (c_{ij}) count the number of single cells with a combination of these attributes, e.g. the top-left cell of the contingency table (c_{00}) counts the number of single cells in group g for which pathway p is enriched. The χ-squared test then tests if being in group g is statistically independent from having a significant result for pathway p. The χ-squared test statistics is defined as

$$\chi = n \cdot \sum_{i}^{2} \sum_{j}^{2} \frac{(\frac{c_{ij}}{n} - p_{ij})^2}{p_{ij}},$$

with p_{ij} as the estimated probability of c_{ij} calculated based on the global distribution. We obtained a p-value for this χ-squared statistic using Boost version 1.71.

6 Marker gene detection

For each group, we compare the gene expression of this group against all other groups in order to find genes that are associated with a specific group. The marker genes for a specific group are calculated as follows: For each group, the expression matrix

is separated into cells belonging to the given group and cells that do not belong to the group. For these two new groups of cells, a Wilcoxon rank-sum test, and an independent-shrinkage t-test is performed using GeneTrail with default parameters.

7 Dimension reduction

In order to visualize the cells, we offer several dimension reductions including UMAP and t-SNE. The t-SNE dimension reduction is calcuated with Seurat3 [7, 8] using the following parameters: In the normalization step of Seurat3, we use “LogNormalize” as normalization method, and 10000 as scaling factor. To reduce the dimensionality of the data set, only the most variable genes are kept as features. To find the most variable genes, we select “vst” as selection method. The number of most variable genes is a parameter of the GeneTrail single cell workflow and defaults to 2000. Additionally, we choose 50 as perplexity. The UMAP dimension reduction is also calculated with Seurat3 using the same parameters and 30 for the n.neighbours parameter.

Finally, we provide another UMAP reduction representation calculated with Monocle3 [9], which is also the basis for the pseudo-time analysis described in the following section. For the Monocle3 analysis, we first reduced the dimension of the gene expression data set to the first 50 principal components and afterwards performed a dimension reduction to the first two UMAP coordinates using standard parameters of Monocle3.

8 Pseudo-time analysis

The pseudo-time analysis offered by GeneTrail is performed using the Monocle3 R package with standard parameters and a dimension reduction as discussed above. The basis for the pseudo-time calculation is a graph learning algorithm that tries to learn the ancestry of the cells. Monocle3 provides the coordinates of the graph vertices in their UMAP reduction space. Therefore, it is currently not possible to change the dimension reduction representation to any other than the UMAP representation of Monocle3 for the pseudo-time plot on our results page. If the graph is generated, the pseudo-time is calculated based on the distance to a selected start cell. In our automatized workflow, we currently do not support the manual selection of a start cell, but we estimate the start cell using a script from the Monocle3 documentation.

9 Enrichment analysis of user-selected groups

On the results page, we offer an on-demand analysis to compare user-selected groups against each other by performing an enrichment analysis. To this end, a user can decide which groups (i.e. cells with the same annotation) should be part of either the sample or the reference set of the enrichment analysis. The annotation can originate from the metadata file or from the clusters that GeneTrail automatically calculated. If more than one group of cells is chosen for the sample (reference) set, the union of the cells in these

groups is taken as sample (reference) set. To ensure mutual exclusiveness of the sample and reference set, the cells that are in both sets are removed.

After the assignment of annotation groups to either the reference or the sample set and an appropriate curation of the reference and the sample set as described above, we perform an enrichment analysis to compare the sample to the reference set.
We compare the expression of the sample and the reference set as follows: For each gene i, we calculate the mean expression value in the sample set m_{si} and the mean expression value in the reference set m_{ri}. A score for each gene is calculated as $s_i = m_{si} - m_{ri}$. Hence, if the expression values for this gene are on average higher (lower) in the sample set than in the reference set, the gene is assigned a positive (negative) score. On the resulting list of scores, we perform two ORAs, one for the highest 1000 positive genes (representing the sample group) and one for the lowest 1000 negative genes (representing the reference group). The ORA is performed using the GeneTrail workflow with default parameters (see Section 4.1 of this supplement for further information on ORA in GeneTrail).

References

[1] Luecken, M. D., and Theis, F. J. (2019). Current best practices in single-cell RNA-seq analysis: a tutorial. Molecular systems biology, 15(6).

[2] Backes, C., Keller, A., Kuentzer, J., Kneissl, B., Comtesse, N., Elnakady, Y. A., ... and Lenhof, H. P. (2007). GeneTrail—advanced gene set enrichment analysis. Nucleic acids research, 35(suppl_2), W186-W192.

[3] Stöckel, D., Kehl, T., Trampert, P., Schneider, L., Backes, C., Ludwig, N., ... and Meese, E. (2016). Multi-omics enrichment analysis using the GeneTrail2 web service. Bioinformatics, 32(10), 1502-1508.

[4] Wagner, G. P., Kin, K., and Lynch, V. J. (2012). Measurement of mRNA abundance using RNA-seq data: RPKM measure is inconsistent among samples. Theory in biosciences, 131(4), 281-285.

[5] Robinson, M. D., and Oshlack, A. (2010). A scaling normalization method for differential expression analysis of RNA-seq data. Genome biology, 11(3), R25.

[6] Smid, M., van den Braak, R. R. C., van de Werken, H. J., van Riet, J., van Galen, A., de Weerd, V., ... and Wilting, S. M. (2018). Gene length corrected trimmed mean of M-values (GeTMM) processing of RNA-seq data performs similarly in intersample analyses while improving intrasample comparisons. BMC bioinformatics, 19(1), 236.

[7] Stuart, T., Butler, A., Hoffman, P., Hafemeister, C., Papalexi, E., Mauck III, W. M., ... and Satija, R. (2019). Comprehensive integration of single-cell data. Cell, 177(7), 1888-1902.

[8] Butler, A., Hoffman, P., Smibert, P., Papalexi, E., and Satija, R. (2018). Integrating single-cell transcriptomic data across different conditions, technologies, and species. Nature biotechnology, 36(5), 411-420.

[9] Cao, J., Spielmann, M., Qiu, X., Huang, X., Ibrahim, D. M., Hill, A. J., ... and Trapnell, C. (2019). The single-cell transcriptional landscape of mammalian organogenesis. Nature, 566(7745), 496-502.

# - Supplement S6 -
GeneTrail 3: Parameters for case studies

1 Time series analysis of activated T-cells

1.1 Data set (GSE136625)

The data set contains gene expression microarrays of CD4+ T cells from the blood of two human donors. The extracted T cells were in vitro activated and expression profiles were created at 2h intervals from 0h-24h. For both donors and each time point 3 replicates were created.

1.1.1 Microarrays and normalization

Gene expression profiles were measured using Agilent-039494 SurePrint G3 Human GE v2 8x60K Microarrays (Cat. no. G4851B, Agilent Technologies, Santa Clara, CA, USA) according to the manufacturer's instructions. Raw expression values were extracted using the Agilent Feature Extraction Software. The limma package was then used for background correction (method="normexp", offset=16) and normalization between the arrays (method=quantile).

1.1.2 Preprocessing

For our analysis, we only considered the gene expression profiles of Donor 1. For each time point, we used the median value to aggregate all replicates.

1.2 Parameters for clustering and enrichment analysis

1.2.1 Filtering

Parameter	Value
Difference between minimal and maximal time points	2.0

1.2.2 Clustering - Step 1

Parameter	Value
Distance measure	Euclidean distance for gradients
Linkage method	Complete linkage
Threshold for cluster	0.8
Minimum number of genes for each cluster	1

1.2.3 Clustering - Step 2

Parameter	Value
Distance measure	Euclidean distance for gradients
Linkage method	Complete linkage
Threshold for super-cluster	0.95

1.2.4 Over-representation analysis (ORA)

Parameter	Value
Minimum number of hits	1
Maximum number of hits	700
Method	Hypergeometric test
P-value strategy	upper-tailed
P-value adjustment	Benjamini-Hochberg
Significance level	0.05

2 Aging processes in mouse microglia cells

2.1 Data set (GSE132042)

Here, we analyze a single cell data set of mouse microglia cells from different brain tissues (cerebellum, cortex, hippocampus and striatum). This dataset is part of a comprehensive single cell transcriptome atlas that was designed to study hallmarks of aging in a large variety of mouse tissues and organs (GSE132042, Tabula Muris Senis Project [1]). The subset of microglia cells contains 8330 gene expression profiles of cells from mice with distinct age groups: 3 month, 18 month and 24 month.

2.2 Parameters

2.2.1 Normalization

Parameter	Value
scRNA-Seq Protocol type	Based on full-length sequencing
Normalization method	log(TPM + 1) (includes length normalization)

2.2.2 Filtering

Parameter	Value
Analyze the X most expressed genes per cell	500

2.2.3 Over-representation analysis (ORA)

Parameter	Value
Minimum number of hits	1
Maximum number of hits	700
Method	Hypergeometric test
P-value strategy	upper-tailed
P-value adjustment	Benjamini-Hochberg
Significance level	0.05

2.2.4 Seurat

Parameter	Value
Number of variable genes	2000

References

[1] Pisco, A. O., Schaum, N., McGeever, A., Karkanias, J., Neff, N. F., Darmanis, S., ... Quake, S. R. (2019). A Single cell transcriptomic atlas characterizes aging tissues in the mouse. bioRxiv, 661728.
